# Identification and expression analysis of *PAL* genes related to chlorogenic acid synthesis in *Vaccinium dunalianum* Wight

**DOI:** 10.3389/fpls.2025.1544303

**Published:** 2025-05-02

**Authors:** Xiuhua An, Guoze Li, Aiyi Chen, Ping Zhao, Yong Ding

**Affiliations:** ^1^ College of Biological Science and Food Engineering/Key Laboratory for Forest Resources Conservation and Utilization in the Southwest Mountains of China, Ministry of Education, Southwest Forestry University, Kunming, China; ^2^ School of Basic Medical Sciences, Baoshan College of Traditional Chinese Medicine, Baoshan, China; ^3^ Constant Carbon Research & Development Department, Hua An Tang Biotech Group, Guangzhou, China

**Keywords:** *Vaccinium dunalianum*, phenylalanine deaminase, chlorogenic acid, gene cloning, tissue expression

## Abstract

Phenylalanine ammonia-lyase (PAL) is the first key enzyme in the metabolic pathway of phenylpropanoid. Chlorogenic acid (CGA) is an important secondary metabolite in the phenylpropanoid metabolic pathway of plants. However, the role of the *PAL* gene in CGA biosynthesis in *Vaccinium dunalianum* Wight is still unclear. In this study, seven *PAL* genes (*VdPAL1*–*VdPAL7*) were cloned and analyzed through full-length transcriptome sequencing combined with reverse transcription polymerase chain reaction (RT-PCR) amplification in *V. dunalianum*. Bioinformatics analysis revealed that the seven *VdPAL* genes contain complete open reading frames (1,860–2,148 bp) encoding hydrophilic proteins of 619–715 amino acids, with molecular weights ranging from 67.44 to 77.70 kDa. The VdPAL proteins contain the PLN02457 domain, with the secondary structures predominantly composed of α-helices and the tertiary structures exhibiting a characteristic “hippocampus” conformation. Phylogenetic analysis grouped these *VdPAL* genes into three subfamilies: *VdPAL1*, *VdPAL3*, and the *VdPAL2/4/5/6/7* cluster. Reverse transcription quantitative polymerase chain reaction (RT-qPCR) analysis showed that the expression levels of the *VdPAL1* and *VdPAL4/5/6/7* genes were significantly higher in both tender and mature leaves, in flower buds, flowers, green and red fruits, and in green and red fruit stems than those of *VdPAL2* and *VdPAL3*. In the same tissue type, except for *VdPAL2*, the expression levels of the other six *PAL* genes were significantly higher in young tissues compared with mature tissues. The *PAL* gene family mediates the biosynthesis of CGA in *V. dunalianum*, with the combined expression of *VdPAL1/2/4/5/6/7* showing a positive correlation with the CGA content across the analyzed tissues. These results provide experimental evidence for understanding the composition, structural characteristics, and biological functions of the *PAL* genes in *V. dunalianum*.

## 1 Introduction

*Vaccinium dunalianum* is a medicinal plant belonging to the genus *Vaccinium* of the family Ericaceae. It is mainly distributed in the southwestern region of China (Yunnan, Guizhou, and Sichuan). The young leaf buds of *V. dunalianum* are traditionally used by the Yi people in Yunnan Province for the preparation of a tea known as “Quezui tea.” The plant is rich in flavonoids, phenolic acids, and other abundant secondary metabolites (Cheng et al., 2022). Prior research works have highlighted the medicinal properties of Quezui tea, including its ability to promote liver detoxification, improve blood circulation, and reduce blood glucose and lipid levels (Zhang et al., 2022; Yang et al., 2023). Previous studies found that the chlorogenic acid (CGA) content of *V. dunalianum* could be as high as 60–108 mg/g (dry weight, DW) (Zhao et al., 2008; Luo et al., 2015), which is close to the highest CGA content in coffee (*Coffea canephora*) (Upadhyay and Mohan Rao, 2013). Thus, *V. dunalianum* is a characteristic resource plant rich in CGA. Recent investigations have identified 15 key structural genes influencing the CGA biosynthesis in *V. dunalianum*, along with three potential transcription factors that may regulate its biosynthesis (Zhang et al., 2024).

CGA is an important phenolic compound formed through the dehydration and condensation of caffeic acid and quinic acid via the phenylpropanoid biosynthesis pathway, including mono-, di-, tri-, tetra-, and mixed esters (Clifford et al., 2017). CGA has multiple biological actions, including free radical scavenging, antioxidant, liver and kidney protection, and sugar and lipid metabolism regulation (Santana-Galvez et al., 2017). While CGA has demonstrated pivotal significance in medical applications, its functional contributions to the food industry are equally noteworthy. Despite being a naturally occurring phytochemical with diverse botanical sources, CGA typically occurs in low concentrations across most plant species, with limited taxa exhibiting an elevated CGA accumulation. Coffee and sorghum are recognized CGA-rich plants (Zeng et al., 2022; Li Y. et al., 2019). CGA is also ubiquitously present in common vegetables and fruits such as potatoes (Peng et al., 2022), apples (Liao et al., 2021), citrus (Yin et al., 2024), and peaches (Su et al., 2023). Previous studies have documented the CGA content in tomato ranging from 0.021 to 0.24 mg/g (DW) (Floare-Avram et al., 2020), while jasmine tea contains 0.057–0.31 mg/g CGA (Vu and Alvarez, 2021). In *Solanum melongena* (eggplant), CGA constitutes the predominant phenolic compound in pulp tissues, with concentrations between 1.4 and 28.0 mg/g (Plazas et al., 2013). Notably, *V. dunalianum* exhibited exceptional CGA accumulation levels of 44.71–97.64 mg/g (DW) (Zhang et al., 2024; Yang et al., 2024). The biosynthesis of CGA primarily relies on three key enzymes: phenylalanine ammonia-lyase (PAL), shikimic acid/quinic acid hydroxyl cinnamyl transferase (HCT), and quinic acid cinnamate hydroxyl transferase (HQT) (Wang et al., 2022). Increased concentrations of these enzymes can greatly increase the concentration of CGA.

PAL is the first key and rate-limiting enzyme in the phenylpropanoid metabolic pathway, catalyzing the deamination of L-phenylalanine (L-Phe) to form *trans*-cinnamic acid. PAL not only participates in numerous physiological activities during plant growth and development but also plays a critical role in plant stress responses (Clifford et al., 2017; Deng and Lu, 2017). *PAL* genes are present in large numbers in plants and, to a lesser extent, in algae and fungi, and the AvPAL protein derived from *Anabaena variabilis* is clinically utilized as a therapeutic agent for phenylketonuria (Fan et al., 2024). Although PAL is absent in animals, including humans, specific bacterial species have been identified to deaminate phenylalanine, producing cinnamic acid via enzymatic activity (Hyun et al., 2011; Hou et al., 2019; Xiang and Moore, 2005). First reported in barley (1961), *PAL* genes typically exist as multi-member families across species, including four *ChPALs* in *Cephalotaxus hainanensis* (He et al., 2020), seven *CsPALs* in *Camellia sinensis* (Chen et al., 2022), 14 *StPALs* in *Solanum tuberosum* (Mo et al., 2022), and nine *OsPALs* in *Oryza sativa* (Gho et al., 2020). Distinct PAL isoforms exhibit functional specificity across divergent branches of the phenylpropanoid pathway. Genetic studies in *Arabidopsis thaliana* have demonstrated severely impaired flavonoid biosynthesis in *AtPAL1/AtPAL2* double-knockout mutants (Rohde et al., 2004). The expression of *CsPAL4* was positively associated with anthocyanin accumulation in *C. sinensis* (Chen et al., 2022). Functional characterization identified *CcPAL2* as a key contributor to CGA accumulation in *C. canephora* (Lepelley et al., 2012), while the heterologous overexpression of *IbPAL1* significantly enhanced the CGA production in *Ipomoea batatas* (Yu et al., 2021). The regulatory involvement of most *StPALs* in *S. tuberosum* responded to high-temperature and drought stresses (Mo et al., 2022). Recent functional studies have confirmed the participation of *CsPAL9* and *CsPAL7* in thermotolerance mechanisms in *Cucumis sativus* (Amjad et al., 2024). By analyzing the transcriptome data from three developmental stages of *V. dunalianum* (i.e., leaf buds, young leaves, and mature leaves), 15 key structural genes, including three *PAL* genes affecting the biosynthesis of CGA in *V. dunalianum*, were identified, as well as three potential transcription factors that may regulate its biosynthesis (Zhang et al., 2024). These findings elucidated the biosynthetic pathway of CGA in *V. dunalianum* and provided evidence for unraveling the regulatory network of CGA biosynthesis. However, the number of *PAL* genes and their sequence characteristics in *V. dunalianum* have not been reported in detail. Moreover, the expression characteristics of the *PAL* genes in the different tissues of *V. dunalianum* and the mechanisms involved in the regulation of CGA synthesis remain unclear.

In this study, we utilized the three-generation full-length transcriptome database of *V. dunalianum* to screen the *PAL* gene sequences. Full-length *VdPAL* sequences were subsequently validated through reverse transcription polymerase chain reaction (RT-PCR) and cloning. In addition, the spatial expression profiles of the *VdPAL* genes, the PAL enzymatic activities, and the CGA accumulation levels were quantified across eight tissues using reverse transcription quantitative PCR (RT-qPCR), spectrophotometry, and HPLC, respectively. Correlative analyses between *VdPAL* expression and CGA content were systematically conducted.

## 2 Materials and methods

### 2.1 Plant material

Different tissues of *V. dunalianum*, including tender leaves (TLs), mature leaves (MLs), flower buds (FBs), blooming flowers (F), immature green fruits (GFs), ripe red fruits (RFs), green fruit stem (GFS, i.e., fruit peduncles during the young green period), and red fruit stem (RFS, i.e., fruit peduncles during the mature red period), were collected from wild plants in Wuding County, Chuxiong Prefecture, Yunnan Province, China (25°45′ N, 102°17′ E) in July 2020. The above tissue samples with three biological replicates were flash-frozen in liquid nitrogen on site and later stored at −80°C in the laboratory.

### 2.2 Experimental methods

#### 2.2.1 RNA extraction and cDNA synthesis

The eight tissue samples (TLs, MLs, FBs, F, GFs, RFs, GFS, and RFS) of *V. dunalianum* were ground in liquid nitrogen. Total RNA was extracted using the OmniPlant RNA Kit (DNase I; Kangwei, Jiangsu, China). RNA purity was measured with a NanoDrop2000 Nucleic Acid Protein Analyzer (Thermo Fisher Scientific, Shanghai, China), and integrity was assessed via 1% agarose gel electrophoresis. Qualified RNA was reverse-transcribed into cDNA using the HiFiScript cDNA Synthesis Kit (Kangwei, Jiangsu, China).

#### 2.2.2 Cloning of *VdPAL* genes

The three-generation full-length transcriptome database (PRJNA1037676) of *V. dunalianum* completed by our research team (Zhang et al., 2024) was used to mine and screen the *PAL* genes. Candidate transcripts were aligned using DNAMAN software to remove duplicates. Conserved domains were verified via NCBI CD-Search, and unreliable transcripts were discarded. Validated *VdPAL* sequences were used to design gene-specific primers (**Supplementary Table S1**). The cDNA samples from TLs were used as templates, and specific primers were used for PCR amplification. The reaction system was 50 μl: PrimeSTAR Max Premix (2×) 25 μl, upstream and downstream primers 1 μl, cDNA 200 ng, and ddH_2_O added to 50 μl. The reaction procedures were as follows: 98°C for 3 min; 98°C for 10 s, 55°C for 15 s, and 72°C for 2 min, 35 cycles; and 72°C for 5 min. The PCR reaction products were detected with 1% agarose gel electrophoresis. The target fragments were purified and ligated into the pClone007 vector for cloning. DNA sequencing was performed by Tsingke Biotechnology Co., Ltd.

#### 2.2.3 Bioinformatics analysis of the *VdPAL* gene family

The sequencing sequences were aligned with the transcriptome sequences using DNAMAN software. The NCBI ORF-Finder program was used to determine the open reading frames (ORFs) of the genes. The deduced amino acid sequences were analyzed for physicochemical properties (i.e., sequence length, isoelectric point, molecular weight, instability index, and hydropathicity) using the ProtParam tool (https://web.expasy.org/protparam/). The TMHMM online program (http://www.cbs.dtu.dk/services/TMHMM/) was used to predict the transmembrane structural domains and to analyze the subcellular localization. The protein secondary structures were predicted with the online software SOPMA (https://npsaprabi.ibcp.fr/), while the protein tertiary structures were predicted with the online software SWISS-MODEL (https://swissmodel.expasy.org/). The amino acid sequences encoded by the target genes were homology compared using the NCBI (https://www.ncbi.nlm.nih.gov/) BLAST program. Conserved motifs and domains were identified using MEME (http://meme-suite.org/tools/meme) and SMART (http://smart.embl-heidelberg.de/), respectively. Phylogenetic trees were constructed with MEGA11 using the neighbor-joining (NJ) method.

#### 2.2.4 Expression analysis of the *VdPAL* genes in different tissues of *V. dunalianum*

Fluorescent quantitative primer pairs (**Supplementary Table S2**) were designed using the NCBI Blast-Primer online tool, and RT-qPCR amplification was performed using cDNA (100 ng) from the eight tissues (TLs, MLs, FBs, F, GFs, RFs, GFS, and RFS) of *V. dunalianum*, with *Vd60S-2* as an internal reference gene. RT-qPCR was performed following the instructions for TB Green Premix Ex Taq II (Tli RNaseH Plus, 2×) (TaKaRa, Beijing, China). Three biological replicates were analyzed per sample, and relative gene expression was calculated using the 2^−ΔΔCt^ method.

#### 2.2.5 Extraction of the crude enzyme solution and determination of the PAL enzyme activity

Sample preparation involved grinding 1 g of fresh tissue samples (TLs, MLs, FBs, F, GFs, and RFs) in liquid nitrogen using a mortar and pestle. Of 0.1 M boric acid buffer (pH 8.8, containing 2 mM of β-mercaptoethanol and 0.1 mM EDTA-Na_2_), 5 ml was added, followed by ice-cold homogenization for 20 min. After centrifugation of the homogenate at 12,000 rpm for 20 min (4°C), the resultant supernatant was collected as crude enzyme extract and stored at 4°C for subsequent analysis. The reaction system consisted of 1 ml of 0.02 M L-Phe, 2 ml of 0.1 M boric acid buffer (pH 8.8) (control group replacement: boric acid buffer for substrate), and 1 ml of the crude enzyme extract. After thorough mixing, the mixture was incubated at 30°C for 30 min, with the reaction terminated by adding 0.2 ml of 2 M HCl. The enzyme activity was quantified by measuring the absorbance at 290 nm, where 1 U was defined as the amount of enzyme required to produce 1 μg cinnamate per milliliter reaction mixture per hour (ΔOD_290_ = 0.01/h). Moreover, the protein content in the crude enzyme extracts from the different *V. dunalianum* tissues was determined using the Coomassie Brilliant Blue G-250 staining method.

#### 2.2.6 Measurement of the CGA content

The eight tissues (TLs, MLs, FBs, F, GFs, RFs, GFS, and RFS) were pre-cooled in a −80°C ultra-low freezer for 12 h. They were then transferred into a vacuum freeze dryer and lyophilized for 32 h under a vacuum of 49 mTorr at −67.2°C. Subsequently, the lyophilized samples were ground using a high-speed grinder (passed through a 40-mesh sieve) and stored at −80°C. The sample was equilibrated in a dryer overnight before determination. A 1.000 g aliquot of each sample was weighed and dissolved in 1.6 ml of 73% methanol. Ultrasonic extraction was performed in a water bath for 19 min, followed by centrifugation at 5,000 rpm for 2 min to obtain the supernatant for further processing. The entire supernatant was transferred into a fresh tube. The extraction was repeated four times for each sample, and the four supernatants were pooled in equal volumes and filtered through a 0.45 μm membrane. Three biological replicates were established for each sample.

Of the CGA, 5.000 mg was precisely weighed using an electronic analytical balance. The standard was dissolved in 73% methanol and diluted to a final volume of 10 ml in a volumetric flask, achieving a concentration of 0.5 μg/μl. The solution was filtered through a 0.45 μm membrane filter. Sample volumes of 1, 2, 4, 8, 16, 25, and 32 μl were injected for analytical detection.

HPLC (Waters 2695, Milford, MA, USA) was used to determine the content of CGA in the different tissues of *V. dunalianum*. Sample separation was achieved with an F90104 CAPCELL PAK C18 MG(S-5) column (250 mm × 4.6 mm, 5 μm), a binary solvent system of 1% aqueous acetic acid (A), and methanol (B). CGA was delivered at a flow rate of 1.00 ml/min, a column temperature of 30°C, an injection volume of 10 μl, and a detection wavelength of 280 nm. The gradient elution procedure consisted of 0.01–5 min to maintain 5% B, 5.01–10 min to maintain 15% B, 10.01–50 min to maintain 65% B, 50.01–55 min to maintain 95% B, and 55.01–70 min to maintain 5% B. The CGA standard (purity ≥98%) was purchased from Shanghai Yuanye Bio-Technology Co., Ltd. Triplicate measurements were performed, and concentrations were calculated from peak areas.

#### 2.2.7 Statistical analyses

Original data were processed using Microsoft Excel 2010. Duncan’s multiple method under one-way ANOVA in SPSS 21.0 software was employed to analyze the significance of the differences in the *VdPAL* gene expression, the PAL enzyme activity, and the CGA content in the different tissues (*p* < 0.05).

## 3 Results and analyses

### 3.1 Identification and cloning verification of the *VdPAL* genes

**Figure 1:**
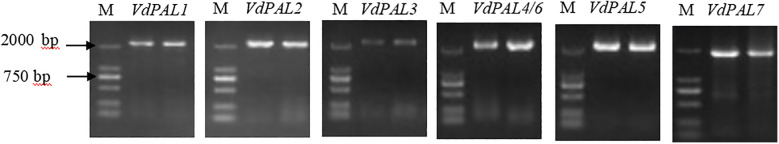
Cloning of *VdPAL* from *Vaccinium dunalianum*.

From the three-generation full-length transcriptome database of *V. dunalianum*, seven VdPAL gene family members (*VdPAL1/2/3/4/5/6/7*) were identified, with sequence accession numbers of OK624452, OK624453, OK624454, OK624455, OK624456, OK624457, and OK624458, respectively. Using tender leaf cDNA as a template, gene-specific primers were designed based on the screened sequences (**Supplementary Table S1**). RT-PCR amplification yielded seven full-length ORFs (1,860–2,148 bp) encoding polypeptides of 619–715 amino acids, which were sequence-verified (**Figure 1**). As summarized in **Table 1**, the VdPAL proteins exhibited molecular weights of 67.44–77.86 kDa and theoretical isoelectric points (p*I*) of 5.90–6.24. Classification as acidic proteins (p*I* < 6.5) was supported by these values. The instability indices (34.16–37.49; instability index <40) indicated inherent protein stability. Comparative analysis revealed that VdPAL7 shares high similarity to other isoforms despite its shorter sequence length. Signal peptide prediction confirmed the cytoplasmic localization for all VdPALs, with TMHMM analysis further excluding transmembrane domains. Hydropathy profiling classified these as hydrophilic proteins, consistent with their cytoplasmic catalytic roles. Collectively, the seven VdPAL isoforms demonstrate conserved physicochemical properties.

**Table 1:** Information on the *VdPAL* family genes and physicochemical properties of the VdPAL proteins.

Gene name	Open reading frame (bp)	Number of amino acids	Molecular weight (kDa)	Negatively charged residue total base (Asp+Glu)	Positive electric charge total number of residues (Arg +Lys)	Isoelectric point	Instability index	Aliphatic index	Hydrophilicity
*VdPAL1*	2,148	715	77.70	83	72	6.00	34.22	93.73	−0.126
*VdPAL2*	2,142	713	77.04	81	71	5.97	35.83	90.43	−0.135
*VdPAL3*	2,132	711	77.86	83	73	6.03	35.82	93.97	−0.156
*VdPAL4*	2,142	713	77.10	81	71	5.97	35.32	90.43	−0.129
*VdPAL5*	2,130	709	76.71	82	71	5.90	34.16	89.97	−0.141
*VdPAL6*	2,142	713	77.07	82	71	5.90	34.69	90.15	−0.129
*VdPAL7*	1,860	619	67.44	69	63	6.24	37.49	90.45	−0.168

### 3.2 Prediction of the secondary and tertiary structures of the VdPAL proteins

**Figure 2:**
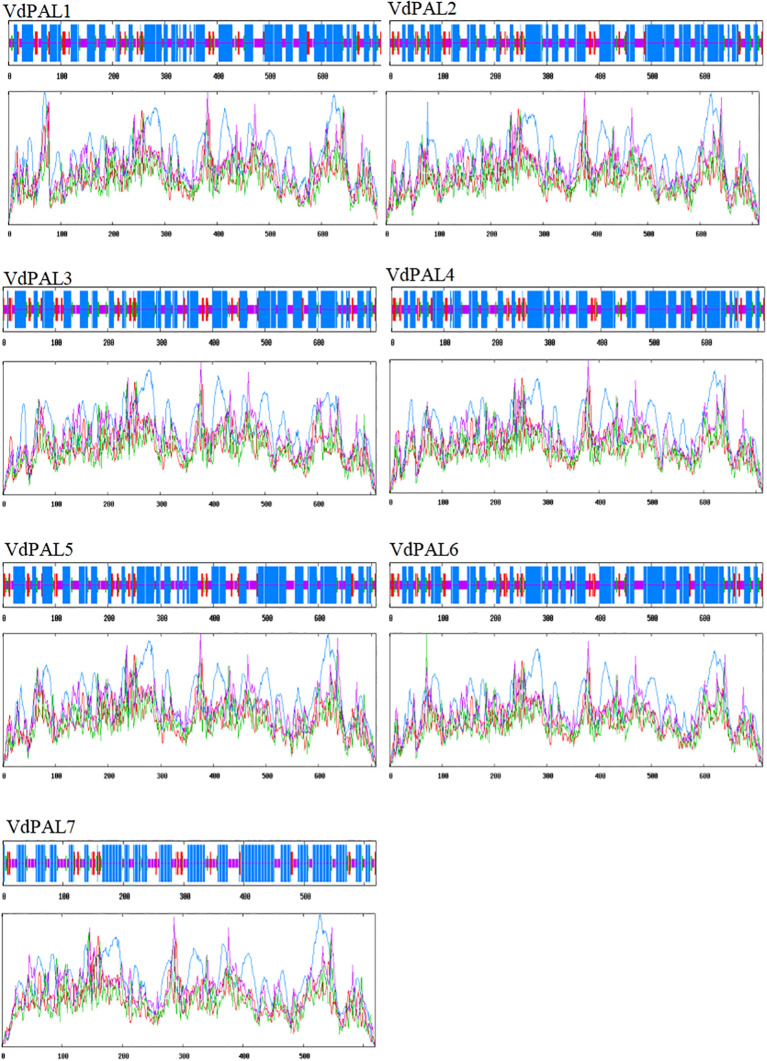
Prediction of the secondary structure of VdPAL. *Blue* represents α-helix, *green* represents β-sheet, *pink* represents random coil, and *red* represents extended strand.

**Figure 3:**
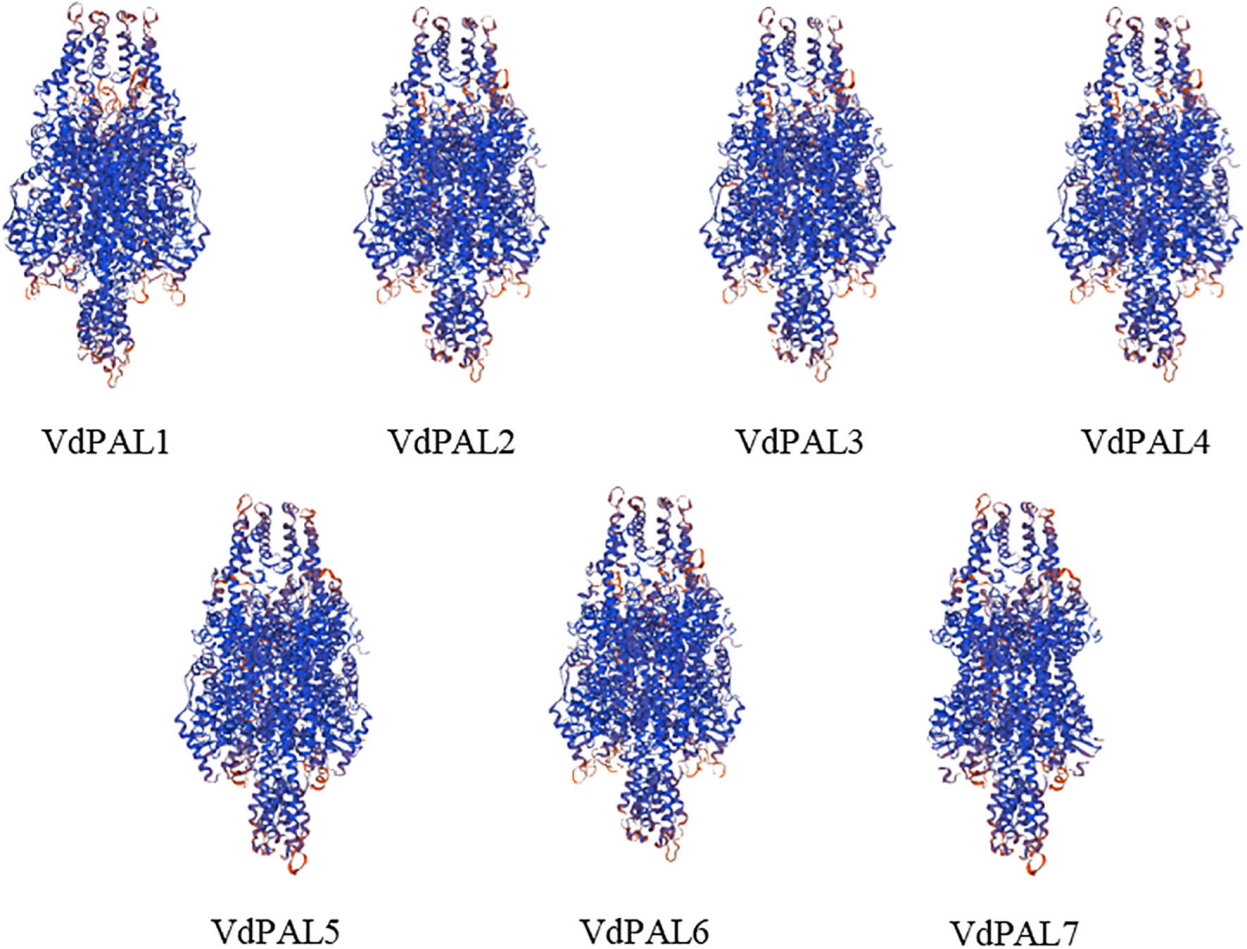
Prediction of the tertiary structure model of VdPAL.

The prediction of the protein secondary structure indicated that the secondary structures of the seven VdPAL proteins are mainly composed of α-helices (54.56%–56.54%), followed by random coils (29.82%–32.02%), with only a small number of β-sheets (5.89%–6.31%) and extended strands (6.95%–8.86%) (**Figure 2**). The Swiss-Model prediction results (**Figure 3**) showed that the tertiary structures of the seven VdPAL proteins all have a typical “hippocampus” shape and exert their biological functions in the form of homotetramers. The structural similarity of these VdPAL proteins suggests that they share similar biological functions.

### 3.3 Protein sequence alignment analysis

**Figure 4:**
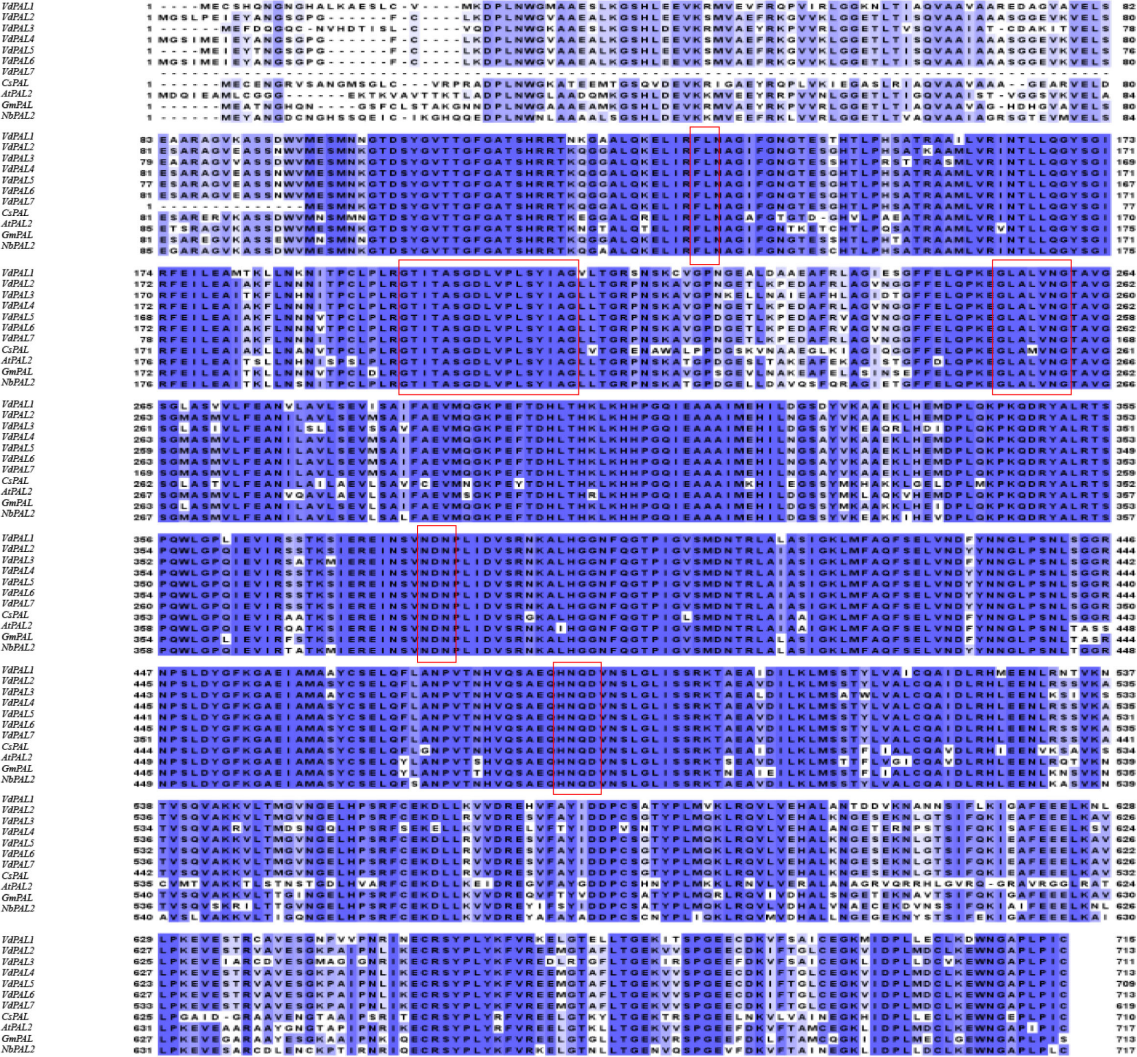
Homology comparison of the amino acid sequences between *VdPAL* and other phenylalanine ammonia-lyase (PAL) protein sequences. *Boxed regions* represent the five substrate recognition sites or catalytic active sites: “FL” residues, “GTITASGDLVPLSYIA” (Ala–Ser–Gly) motif, “GLALVNG,” “NDN”, and “HNQD.”.

Protein sequence homology comparison based on BLAST showed that the VdPAL proteins encoded by the seven *VdPAL* genes cloned here have sequence homology ranging from 83.61% to 93.99% with the OsPAL (*O. sativa*, CAA61198.1), AtPAL2 (*A. thaliana*, AT3G53260), GmPAL (*Glycine max*, CAA37129.1), and NbPAL2 (*Nicotiana benthamiana*, Niben101Scf03712g 01008.1) proteins. Multiple sequence alignment of the aforementioned 11 PAL proteins was generated using the online software Clustal Omega (**Figure 4**). The alignment revealed that the seven VdPAL proteins had a high degree of amino acid sequence conservation among themselves and with the PAL proteins from other species. In addition, they contain the PAL active center sequence GTITASGDLVPLSYVAG, which can catalyze the conversion of L-Phe to *trans*-cinnamic acid.

The ASG tripeptide (Ala–Ser–Gly) in the MIO (3,5-dihydro-5-methylidene-4H-imidazole-4-one) domain was known to be present in the active site residues for the substrate binding and catalysis of the MIO autocatalytic domain formation. In addition, the conserved “FL (Phe–Leu)” dipeptide was also found to be present in VdPALs, with the “FL” residues playing an important role in the substrate specificity of the PAL enzyme. Hence, the presence of the PAL protein finger motif tag (ASG) and “FL” residues is important for VdPALs’ consumption of L-Phe as their sole substrate. The BLAST results showed that seven VdPALs also contain PAL conserved catalytic active sites, namely, “GLALVNG” at positions 262–269 amino acids, “NDN” at positions 389–392 amino acids, and “HNQD” at positions 493–497 amino acids (**Figure 4**). These structural features predicted conserved catalytic activity among the seven VdPAL isoforms.

### 3.4 Phylogenetic analysis of the VdPAL proteins

**Figure 5:**
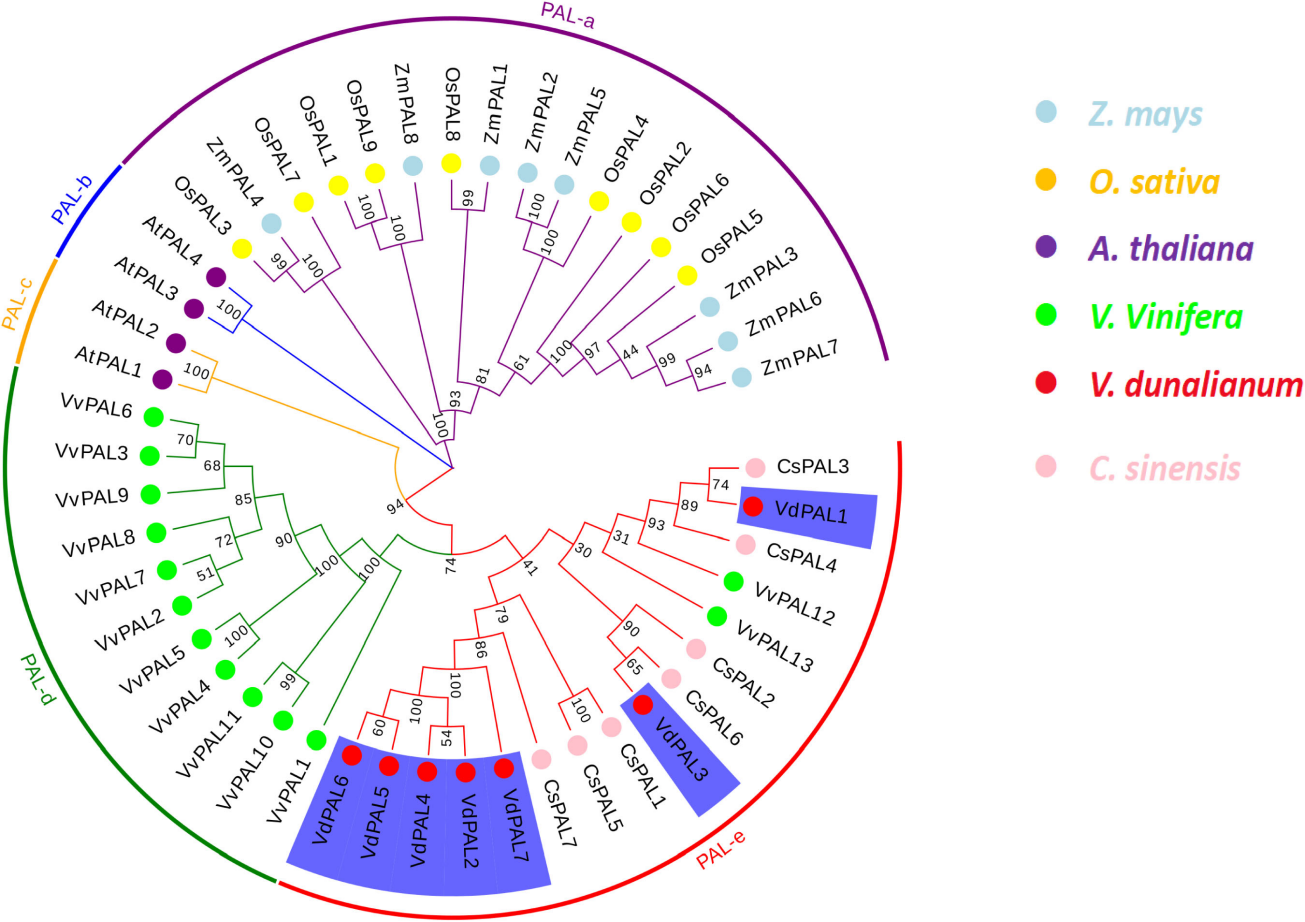
Phylogenetic tree of phenylalanine ammonia-lyase (PAL) from *Vaccinium dunalianum*, *Oryza sativa*, *Arabidopsis thaliana*, *Zea mays*, *Vitis vinifera*, and *Camellia sinensis*. Multiple sequence alignment and phylogenetic tree analyses were conducted using MEGA (V.11.0) with the neighbor-joining (NJ) method (1,000 replicates). The phylogenetic tree was constructed based on the full-length protein sequences.

In order to evaluate the evolutionary relationship of the *VdPAL* genes, phylogenetic analysis was performed on 48 PAL proteins, including seven *VdPALs* from *V. dunalianum*, four *AtPALs* from *A. thaliana*, nine *OsPALs* from *O. sativa*, seven *CsPALs* from *C. sinensis*, eight *ZmPALs* from *Zea mays*, and 13 *VvPALs* from *Vitis vinifera*. The phylogenetic tree constructed using the NJ method based on MEGA11.0 software showed that 48 *PAL* genes were grouped into five *PAL* gene clusters, namely, PAL-a, PAL-b, PAL-c, PAL-d, and PAL-e. These clusters contained 17, 2, 2, 11, and 16 *PAL* gene members, respectively (**Figure 5**). The seven *VdPAL* genes were grouped into the PAL-e cluster according to their phylogenetic positions, but were grouped into different branches of the phylogenetic tree. The *VdPAL2*, *VdPAL4*, *VdPAL5*, *VdPAL6*, and *VdPAL7* genes were clustered in one branch and shared this branch with the *CsPAL1*, *CsPAL5*, and *CsPAL7* genes. *VdPAL1* and *VdPAL3* were clustered in separate branches. *VdPAL1* was clustered in one branch with *CsPAL3* and *CsPAL4*, while *VdPAL3* was clustered in another branch with *CsPAL6* and *CsPAL2*. These results showed that the *PAL* genes from *V. dunalianum* and *C. sinensis* are the most closely related in evolution.

### 3.5 Conserved structural domain and motif analysis of *VdPALs*

**Figure 6:**
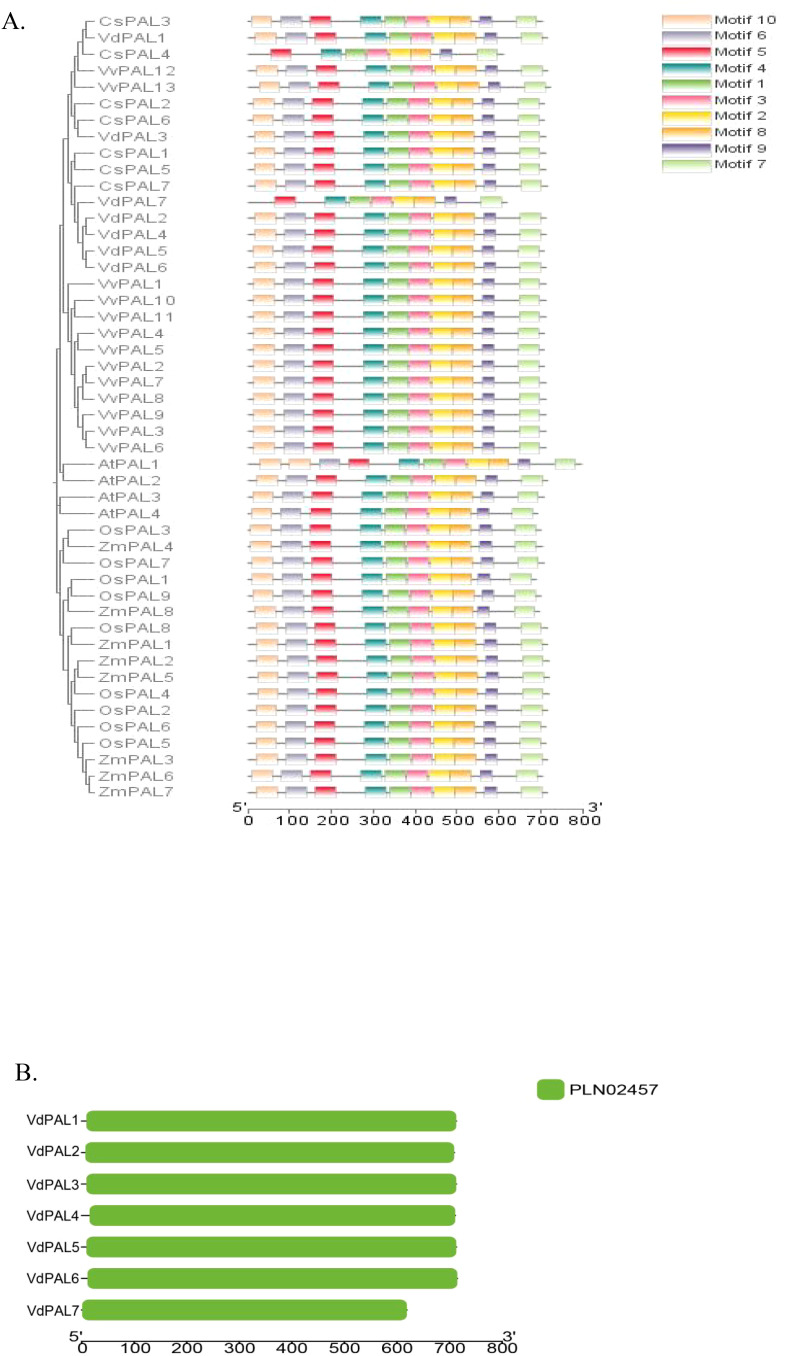
**(A)** Distribution of the conserved motifs in the phenylalanine ammonia-lyase (PAL) proteins from *Vaccinium dunalianum*, *Camellia sinensis*, *Arabidopsis thaliana*, and *Oryza sativa*. **(B)** Analysis of the conservative structures in the VdPAL proteins.

In order to understand the diversity of the protein structure of PAL, the MEME program was used to predict putative conserved motifs within the PAL family of *V. dunalianum*, *O. sativa*, *A. thaliana*, *Z. mays*, *V. vinifera*, and *C. sinensis*. A total of 10 conserved motifs were identified (**Figure 6A**). The results showed that the motif distributions of most PAL proteins were similar. Interestingly, both the VdPAL7 protein in *V. dunalianum* and the CsPAL4 protein in *C. sinensis* showed identical motif patterns, with motifs 10 and 6 absent. Consistent with evolutionary expectations, the closely related genes demonstrated comparable conserved motif distributions. The structural domain positions were determined using CD-Search on the NCBI website. As shown in **Figure 6B**, all seven VdPAL proteins contain the complete PLN02457 domain at distinct positions.

### 3.6 Tissue expression characteristics of the *VdPAL* genes

**Figure 7:**
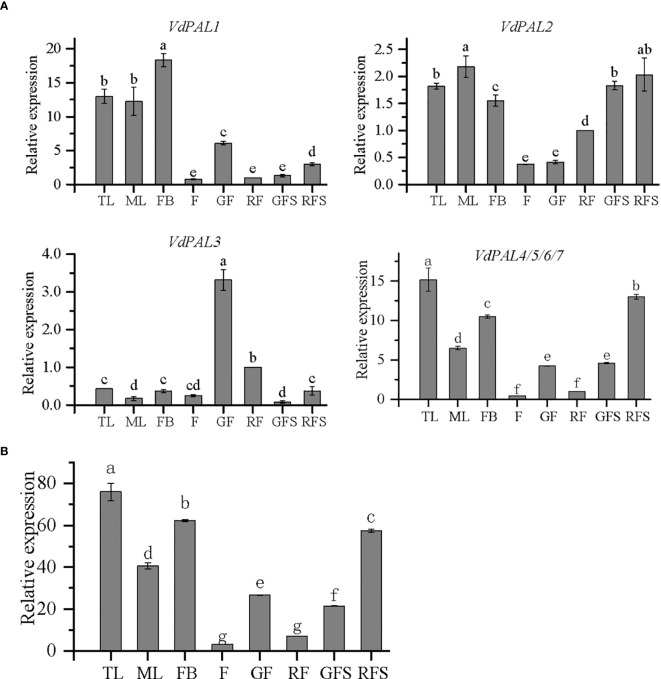
**(A)** Relative expression of *VdPAL* in eight different tissues from *Vaccinium dunalianum*. **(B)** Total expression of the *VdPAL* gene in eight different tissues from *V. dunalianum*. Different lowercase letters indicate significant differences in the different tissues (*p* < 0.05). The error bar is represented by standard deviation. TL, tender leaves; ML, mature leaves; FB, flower buds; F, blooming flowers; GF, immature green fruits, RF, ripe red fruits; GFS, green fruit stem (fruit peduncles during the young green period); RFS, red fruit stem (fruit peduncles during the mature red period).

As shown in **Figure 7A**, the expression of seven *VdPAL* genes at the RNA transcription level was determined in the eight examined tissues of *V. dunalianum*. However, different *VdPAL* genes exhibited distinct tissue-specific expression patterns. In the same tissue, the expression levels of the *VdPAL1* and *VdPAL4/5/6/7* genes were significantly higher than those of the *VdPAL2* and *VdPAL3* genes. Moreover, the expression levels of individual *VdPAL* genes varied markedly across tissues. The expression level of the *VdPAL3* gene in GF and RG tissues was significantly higher than that in the other six tissues (*p* < 0.05), while the expression levels of the other six *VdPAL* genes in TL, ML, and FB tissues were relatively higher than those in the other five tissues (*p* < 0.05). The expression level of the *VdPAL1* gene was the highest in FBs and the lowest in F and RFs. Similarly, the expression levels of the *VdPAL4/5/6/7* genes showed the highest values in TLs, but were the lowest in F and RFs. On the other hand, the expression level of the *VdPAL2* gene peaked in highest in MLs and lowest in F and GFs. Lastly, the expression level of the *VdPAL3* gene exhibited the highest value in GFs, whereas it was the lowest in GFS. As shown in **Figure 7B**, the order of the total gene expression in the eight tissues ranked from the highest to the lowest was: TL > FB > RFS > ML > GF > GFS > RF > F. The gene expression in TL tissue was the highest, while that in F tissue was the lowest. In addition, the gene expression progressively decreased in the leaf and flower tissues during tissue development and the increase of maturity, while the expression in the fruit stem tissue showed an opposite trend with increasing maturity.

### 3.7 Crude enzyme activity of PAL in different tissues of *V. dunalianum*

**Figure 8:**
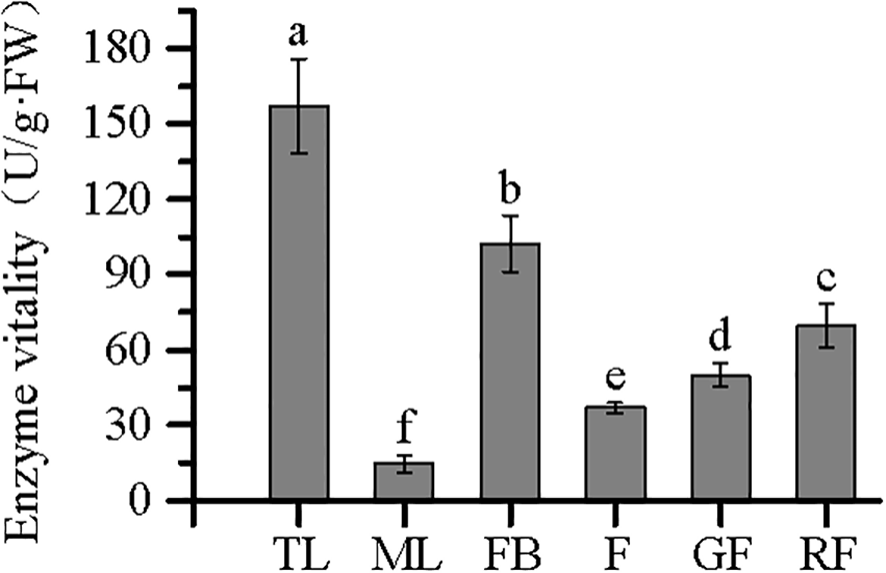
Enzyme viability of phenylalanine ammonia-lyase (PAL) in different tissues from *Vaccinium dunalianum*. Different lowercase letters represent significant differences in the different tissues (*p* < 0.05). The error bar is represented by standard deviation. TL, tender leaves; ML, mature leaves; FB, flower buds; F, blooming flowers; GF, immature green fruits, RF, ripe red fruits.

Owing to the limited quantity of fruit stem tissue samples collected, corresponding enzyme activity data were unavailable; therefore, the analyses were restricted to the remaining six tissues. Comparison of the enzyme activities among tissues revealed significant differences in PAL activity (*p* < 0.05). Specifically, PAL activity was higher in TLs than in MLs, higher in FBs than in F, and higher in mature RFs than in incompletely mature GFs (**Figure 8**). Furthermore, as showed in **Table 2**, the protein concentration was highest in TL, showing a significant difference compared to the other five tissues (*p* < 0.05). The protein concentration in ML ranked second. The protein concentration in FB and GF showed comparable concentrations with no significant difference (*p* > 0.05). The lowest protein concentration was observed in RF.

**Table 2:** PAL enzyme viability and protein concentration in crude fluid from *Vaccinium dunalianum*.

Organization	Enzyme viability (U/g·FW)	Protein concentration (mg/g)
TL	156.99 ± 18.69 a	4.33 ± 0.06 a
ML	14.42 ± 3.45 f	3.19 ± 0.02 b
FB	102.29 ± 11.33 b	2.59 ± 0.07 d
F	36.89 ± 2.02 e	2.90 ± 0.09 c
GF	50.10 ± 4.72 d	2.66 ± 0.21 d
RF	69.58 ± 8.60 c	0.97 ± 0.05 e

Different lowercase letters indicate significant differences in the different tissues (p < 0.05). The error bar is represented by standard deviation.

### 3.8 Content of CGA in different tissues of *V. dunalianum*

**Figure 9:**
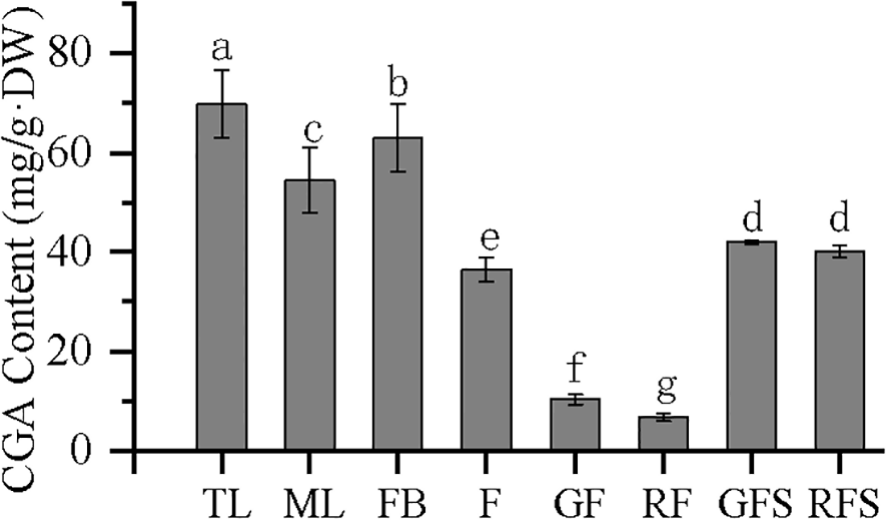
Chlorogenic acid (CGA) content in the different tissues of *Vaccinium dunalianum*. Different lowercase letters represent significant differences in the different tissues (*p* < 0.05). The error bar is represented by standard deviation. TL, tender leaves; ML, mature leaves; FB, flower buds; F, blooming flowers; GF, immature green fruits, RF, ripe red fruits; GFS, green fruit stem (fruit peduncles during the young green period); RFS, red fruit stem (fruit peduncles during the mature red period).

The CGA content in the eight different tissues is shown in **Figure 9**, indicating that CGA accumulates in different tissues of *V. dunalianum*. However, significant differences in the CGA content were observed among the tissues. The order of CGA content, from high to low, was as follows: TL > FB > ML > GFS > RFS > F > GF > RF. The CGA contents in TL and FB tissues were as high as 69.87 and 63.03 mg/g·DW, respectively, while those in RF and GF tissues were only 6.76 and 10.31 mg/g·DW, respectively. In addition, in leaf, flower, and fruit tissues, the CGA content in TL was significantly higher than that in ML, the CGA content in FB was significantly higher than that in F, and the CGA content in GF was significantly higher than that in RF. In fruit stem tissues, the CGA content in GFS was higher than that in RFS, but the difference was not statistically significant. These results indicate that CGA was gradually reduced or metabolized in the same tissue with the occurrence of tissue development and the increase of maturity.

### 3.9 Correlation analysis of the CGA content with *VdPAL* gene expression and VdPAL activity

The correlation between the expression levels of the *VdPAL* genes and the CGA contents in the different tissues of *V. dunalianum* was analyzed. The results showed that the correlation coefficients of the expression of *VdPAL1*, *VdPAL2*, and *VdPAL4/5/6/7* with CGA content were 0.701, 0.653, and 0.710, respectively. Therefore, the expression of *VdPAL1/2/*4/5/6/7 in tissues was significantly positively correlated with the CGA content (*p* < 0.05), indicating that the higher the expression levels of these six *VdPAL* genes in these tissues, the more CGA present in the tissues. In contrast, the expression of *VdPAL3* in tissues was significantly negatively associated with the CGA content (*p* < 0.05), with a correlation coefficient of −0.647, reflecting that the lower the expression of *VdPAL3* in the tissues, the higher the CGA content.

**Figure 10:**
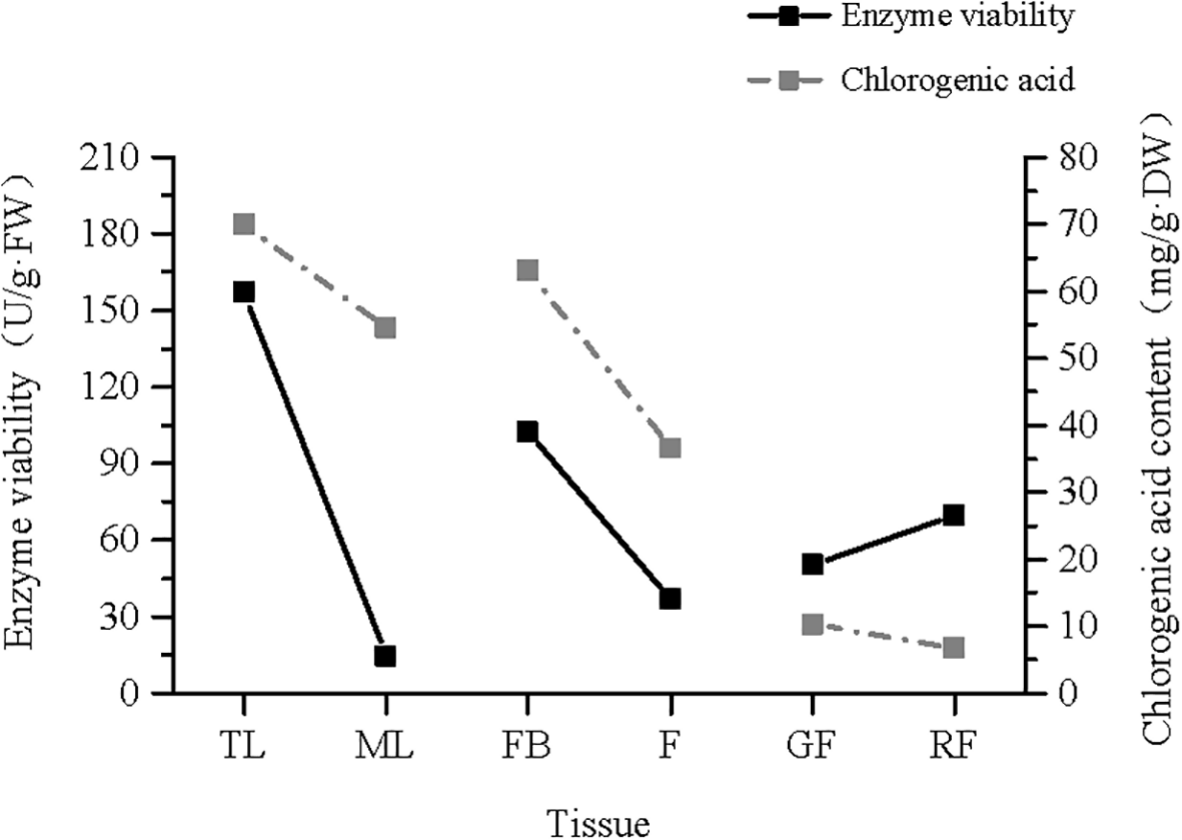
Trend line of the phenylalanine ammonia-lyase (PAL) enzyme ratio and chlorogenic acid (CGA) content in the different tissues of *Vaccinium dunalianum*. TL, tender leaves; ML, mature leaves; FB, flower buds; F, blooming flowers; GF, immature green fruits, RF, ripe red fruits.

The trend diagram of the PAL enzyme activity and CGA content in the different tissues of *V. dunalianum* (**Figure 10**) showed that the CGA content and the PAL enzyme activity were positively correlated in leaf tissues. With increasing leaf tissue maturity, the PAL enzyme activity and CGA content also decreased. The same changes were observed in flower tissues. However, the opposite pattern was observed in the fruit tissue, where both the PAL enzyme activity and the CGA content decreased as the leaf tissue maturity increased.

## 4 Discussion

### 4.1 Identification and phylogenetic analysis of the *VdPAL* genes

PAL plays an important bridging role between the primary and the secondary metabolism in plants and is also a key rate-limiting enzyme in the phenylpropanoid metabolic pathway, which is widely involved in the formation of CGA, lignin, flavonoids, and other compounds. The *PAL* gene family contributes to plant defense against biotic and abiotic stresses, and PAL is a key enzyme in the production of antimicrobial compounds such as plant antitoxins (Tuladhar et al., 2021). To date, comprehensive genome-wide analyses of the *PAL* genes have been reported in many model and non-model plants. For example, a total of nine *OsPAL* genes have been identified in *O. sativa* (Gho et al., 2020), 14 *StPAL* genes in *S. tuberosum* (Mo et al., 2022), 37 *TaPAL* genes in *Triticum aestivum* (Rasool et al., 2021), and three *FpPAL* genes in *Ferula pseudalliacea* (Shahidi et al., 2024). The number of PAL family members may be associated with the metabolic diversity and ecological niche of various plant species, offering valuable insights into their characteristics. Currently, there is limited understanding of the sequence, structure, function, and expression of the *PAL* genes in *V. dunalianum*. In this study, seven *VdPAL* genes containing full-length ORFs were cloned from *V. dunalianum* for the first time, and these gene sequences were registered in the NCBI database. The *VdPAL* genes encoded 619–715 amino acids in *V. dunalianum*, which was similar to the length of the amino acids in the PALs of tea plants and rice (Chen et al., 2022; Gho et al., 2020). The secondary structure of *VdPAL* was mainly dominated by α-helices, which was similar to the PALs of orchids and oilseed rape (Vishwakarma et al., 2023; Zhang et al., 2023). The three-dimensional structures of the VdPAL proteins showed a typical “hippocampus” shape, and they contain the PLN02457 and PAL–HAL domains, indicating that the PAL of *V. dunalianum* is a typical phenylalanine deaminase and belongs to the PAL protein family. The protein sequence alignment analysis indicated that VdPALs share high identity with each other and have a conserved motif “GTITASGDLVPLSYIA” (Ala–Ser–Gly) and the MIO autocatalytic domain (He et al., 2020). The VdPAL proteins have no transmembrane junction structural domains and are present in the cytoplasm, which is in agreement with the results of the *PAL* gene family proteins in *Epimedium pubescens* (Xu et al., 2024). It was also predicted that VdPALs are hydrophilic proteins that are more active in a slightly acidic environment.

### 4.2 Phylogenesis and structure domain analysis of VdPALs

In most plants, the *PAL* genes can be classified into two or three different subgroups or taxa (Zhang and Liu, 2015; Barros and Dixon, 2020). By constructing an NJ tree, phylogenetic analysis showed that the *CsPAL* genes of tea plants formed four groups, and this result was in line with the *PAL* gene family in wheat and sugarcane (Rasool et al., 2021; Wu et al., 2024). Compared with other angiosperms, *VdPAL* has more groups than walnut and citrus (Yan et al., 2019; Wei et al., 2023) and can be divided into five groups, with group a consisting of rice and maize, groups b and c consisting of *A. thaliana*, group d consisting of grape, and group e consisting of *V. dunalianum* and tea plants. These findings showed a similar group distribution of orthologs, suggesting that the *VdPAL* genes are evolutionarily conserved. Furthermore, the results of the phylogenetic analyses were further supported by the conserved motif analyses. In the PAL family, most conserved motifs are critical, including motifs 1–6 and 8, and they may give PAL unique functions, which warrant further investigation. Notably, VdPAL7 differs from the other six VdPAL proteins in that it lacks motifs 6 and 10, suggesting that it may have a unique function within the VdPAL family. The results of the phylogenetic tree analysis showed that the *PAL* gene of *V. dunalianum* is more closely related to tea plants than to rice and maize. The current study on the *PAL* gene family in *V. dunalianum* could not explain the regulatory role of this gene family on the growth, development, and metabolic regulation of *V. dunalianum*. Therefore, further investigation is required in this regulatory process.

### 4.3 Expression profiles of *VdPALs*

Although the proteins encoded by the seven *VdPAL* genes perform the same PAL enzyme function, the expression profiles observed across tissues and tissue maturity levels were not identical, suggesting that the expression of the *PAL* genes is tissue-specific. Previous studies have shown that the expression of *SvPAL* in the leaves of *Salix babylonica* was significantly higher compared with that in other tissues, and the expression of *SvPAL* decreased with the increase in leaf maturity (Gao et al., 2023). In *Artemisia annua*, the expression of *AaPAL* peaked in young leaves, followed by flower buds, and was minimal in the roots (Zhang et al., 2016). Most of the *CsPAL* family members showed high transcription levels in buds and young leaves, whereas all family members had extremely low transcription levels in old leaves (Chen et al., 2022). In this study, most of the *VdPAL* family members showed high transcript levels in TLs and FBs, and all family members had extremely low transcription levels in mature tissues. In contrast, the transcript levels of *PbPAL1* and *PbPAL2* in pear (*Pyrus bretschneideri*) were higher in the roots and stems than in the leaves and buds (Li H. et al., 2019). Factors such as the developmental stages of *V. dunalianum*, the sampling methods, and species specificity could have affected the experimental results. Despite these differences, the high levels of transcription in TLs and FBs suggest that most of these genes (*VdPAL1*, *VdPAL4*, *VdPAL5*, *VdPAL6*, and *VdPAL7*) are involved in the regulation of leaf vegetative growth and sexual reproduction, such as FB development.

### 4.4 Correlation analysis

The HPLC analysis revealed significant variations in the CGA content across *V. dunalianum* tissues, peaking in TLs and reaching minimal levels in RFs. The CGA content changed gradually with fruit development. For example, the content of CGA gradually decreased during the fruit development of pear (*Pyrus sinkiangensis*) in Xinjiang (Wen et al., 2022), while that in peach (*Prunus persica*) showed an increasing trend followed by a decrease with fruit development (Su et al., 2023). With the increase of tissue maturity, the CGA content in *V. dunalianum* showed a downward trend, which may be due to CGA acting as a secondary metabolite, which can also be used as a substrate to generate downstream secondary metabolites, such as lignin and flavonoids (Clifford et al., 2017). In addition, the accumulation of secondary metabolites in the early stage can help plants improve their resistance to the environment and insect pests and to carry out a nitrogen source reserve to ensure their own growth and development (De-la-Cruz Chacón et al., 2013). When the research needs to obtain the active ingredients of a large number of medicinal plants, it should be determined according to the status of the richest cumulative active ingredients. Therefore, selection of the leaf buds of *V. dunalianum* and making “Quezui tea” without using other tissues are scientifically based.

For the mechanism of CGA accumulation in cultivated and wild apples, it was found that the correlations of the expression of *MdPAL3* and *MdHQT* with CGA were 0.8237 and 0.9775, respectively, with the deduced conclusion being that *MdPAL3* and *MdHQT* are the key genes controlling the accumulation of CGA in apple fruits (Liao et al., 2021). In the present study, the expression profile showed that *VdPAL1/4/5/6/7* were higher in TLs and BFs and that they positively regulated the content of CGA, indicating that *VdPAL1/4/5/6/7* may be involved in the regulation of the biosynthesis of CGA in *V. dunalianum*.

In *V. dunalianum*, the PAL enzyme activity in young tissues (TLs and FBs) was higher than that in mature tissues (MLs and F), and PAL activity was positively correlated with the CGA content. In tea leaves, the PAL activity decreased significantly with the maturity of leaves, and a close correlation with catechin accumulation was observed (Samanta et al., 2017). However, red fruits with high ripeness showed higher activity than green fruits, but the CGA content decreased. This phenomenon is mainly due to the ripening process of the fruit of *V. dunalianum*, during which a large amount of anthocyanins and other substances need to be accumulated. The high activity of the PAL enzyme helps produce such secondary metabolites, and CGA, as a precursor for the synthesis of anthocyanins, is also consumed, so that there is an inverse relationship between the activity of the PAL enzyme and the content of CGA in the fruit tissues.

## 5 Conclusion

In this study, seven *VdPAL* genes were cloned and functionally characterized from *V. dunalianum* for the first time, including analyses of the sequence alignment, subcellular localization, phylogenetic relationships, protein structure, and motif composition. Interestingly, the seven *VdPAL* genes exhibited a remarkably high degree of similarity, suggesting that they may have undergone repeated duplication events in their evolutionary history. Despite their structural similarities, the expression patterns of these genes in different tissues exhibited significant variations. These differences in expression suggest the existence of tissue-specific regulatory mechanisms. Notably, the expression of *VdPAL1/2/4*/*5*/*6*/*7* was significantly positively correlated with the CGA content, while that of *VdPAL3* was significantly negatively correlated with the CGA content. These results indicate that the *VdPAL1/2/4/5/6/7* genes are involved in CGA biosynthesis. Subsequently, functional validation of the genes with significant differences in the relative expression of *V. dunalianum* will be conducted, and the effects of the *VdPAL* gene family members on CGA biosynthesis will be further investigated.

## Data Availability

The datasets presented in this study can be found in online repositories. The names of the repository/repositories and accession number(s) can be found in the article/**Supplementary Material**.
