# Supplementary figures and images for: Identification and expression analysis of PAL genes related to chlorogenic acid synthesis in Vaccinium dunalianum Wight
Source: Front Plant Sci. 2025 May 2;16:1544303. doi: 10.3389/fpls.2025.1544303 (PMC12081449; doi:10.3389/fpls.2025.1544303)

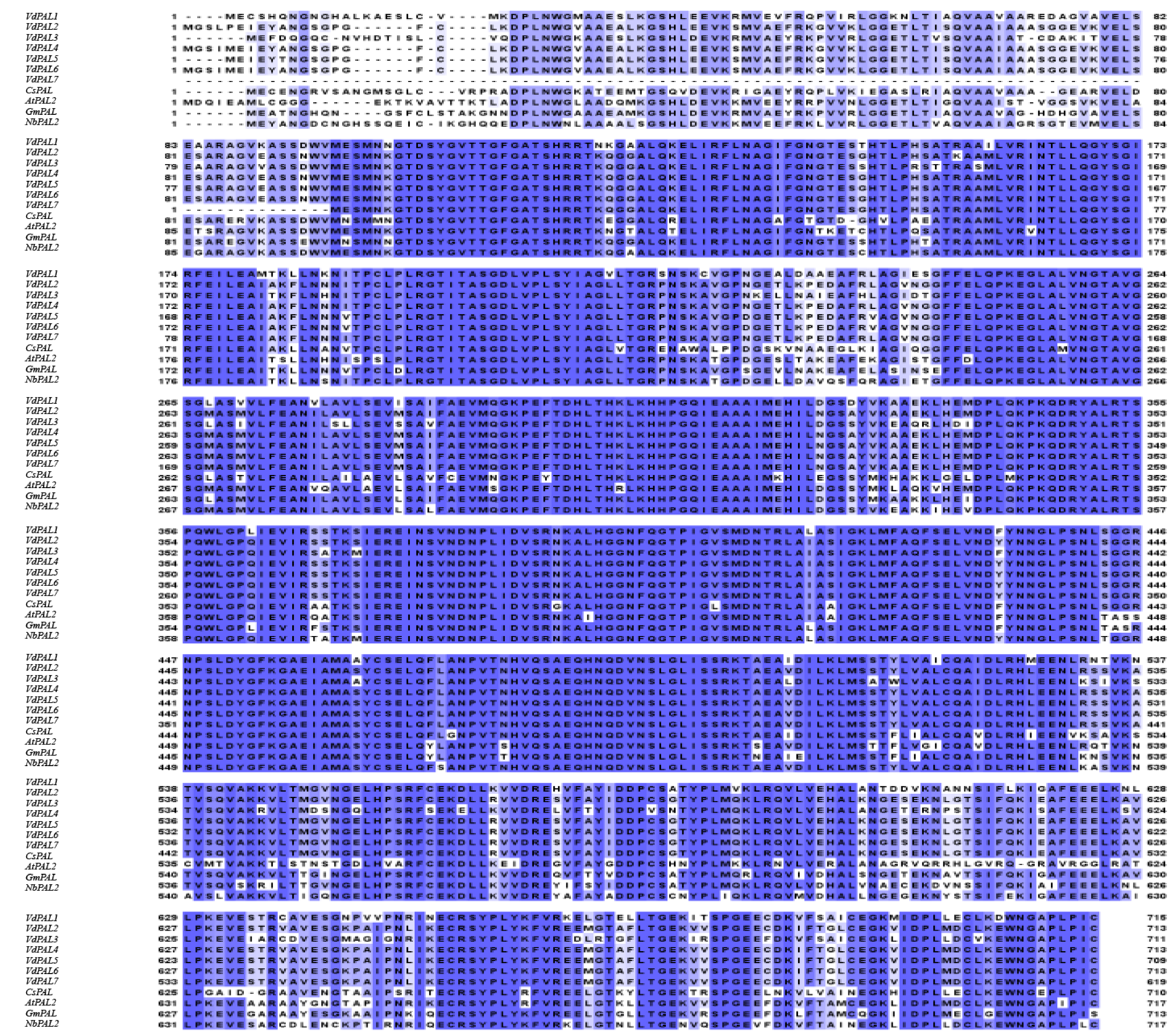

Supplement: Supplementary file 1 [file DataSheet1.zip › picture/blast.png]

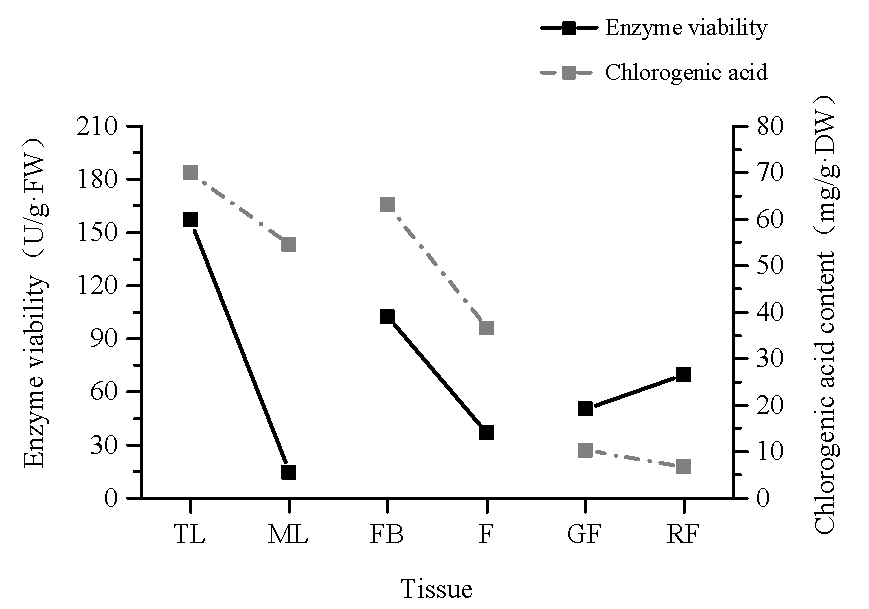

Supplement: Supplementary file 1 [file DataSheet1.zip › picture/correlation between PAL activity and CGA content.png]

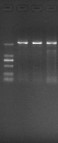

Supplement: Supplementary file 1 [file DataSheet1.zip › picture/gel electrophoresis/PAL1 PCR amplification DL2000.jpg]

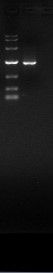

Supplement: Supplementary file 1 [file DataSheet1.zip › picture/gel electrophoresis/PAL12 gel recovery DL10000.jpg]

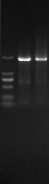

Supplement: Supplementary file 1 [file DataSheet1.zip › picture/gel electrophoresis/PAL12 PCR amplification DL2000.jpg]

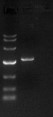

Supplement: Supplementary file 1 [file DataSheet1.zip › picture/gel electrophoresis/PAL1gel recovery DL10000.jpg]

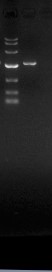

Supplement: Supplementary file 1 [file DataSheet1.zip › picture/gel electrophoresis/PAL2 gel recovery DL10000.jpg]

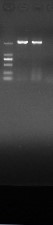

Supplement: Supplementary file 1 [file DataSheet1.zip › picture/gel electrophoresis/PAL2 PCR amplification DL2000.jpg]

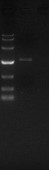

Supplement: Supplementary file 1 [file DataSheet1.zip › picture/gel electrophoresis/PAL3 gel recovery DL10000.jpg]

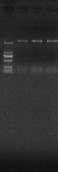

Supplement: Supplementary file 1 [file DataSheet1.zip › picture/gel electrophoresis/PAL3 PCR amplificationDL2000.jpg]

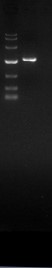

Supplement: Supplementary file 1 [file DataSheet1.zip › picture/gel electrophoresis/PAL4_6 gel recovery DL10000.jpg]

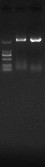

Supplement: Supplementary file 1 [file DataSheet1.zip › picture/gel electrophoresis/PAL4_6 PCR amplification DL2000.jpg]

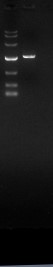

Supplement: Supplementary file 1 [file DataSheet1.zip › picture/gel electrophoresis/PAL5 gel recovery DL10000.jpg]

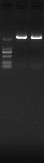

Supplement: Supplementary file 1 [file DataSheet1.zip › picture/gel electrophoresis/PAL5 PCR amplification DL2000.jpg]

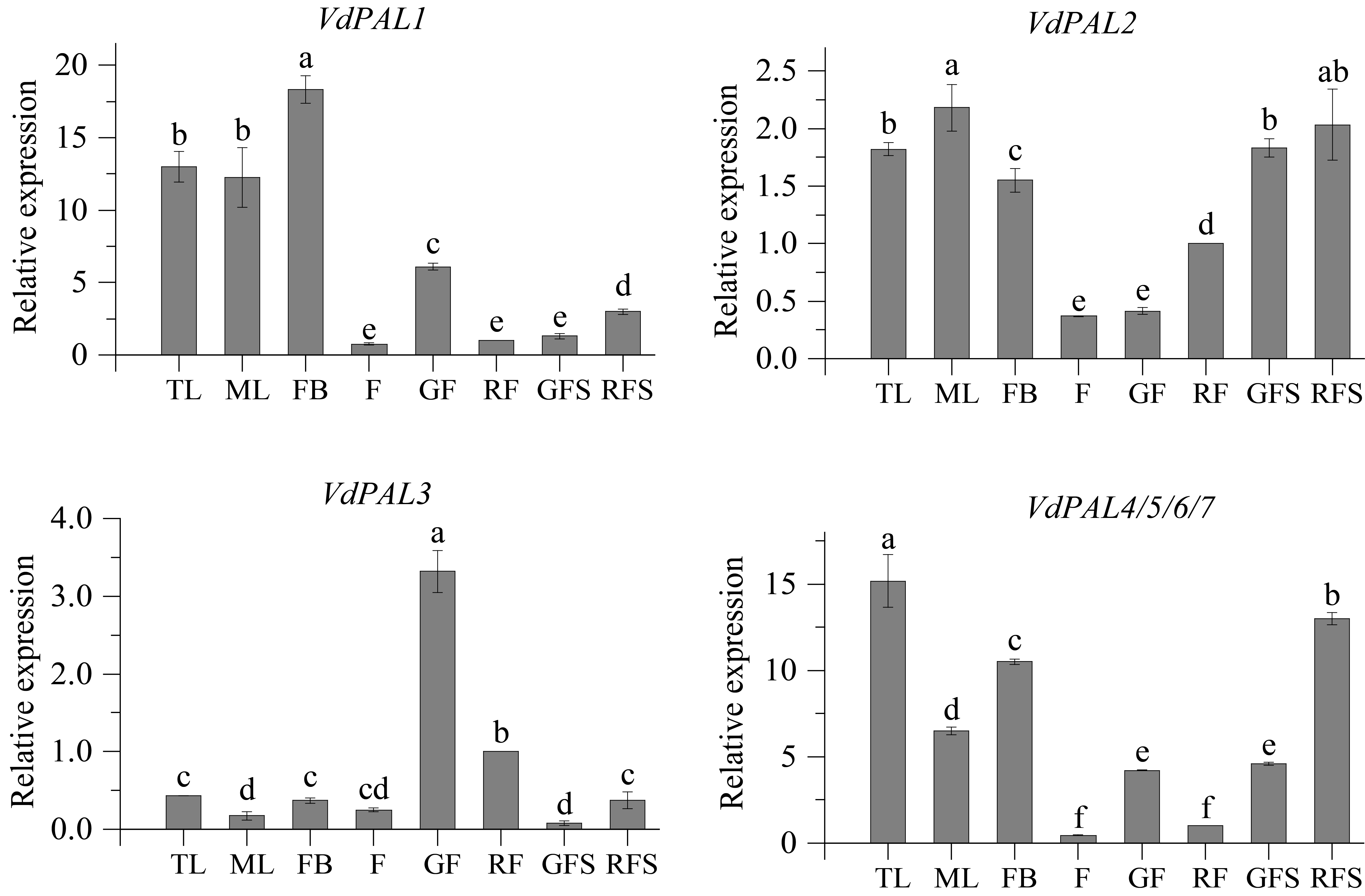

Supplement: Supplementary file 1 [file DataSheet1.zip › picture/gene expression quantity.png]

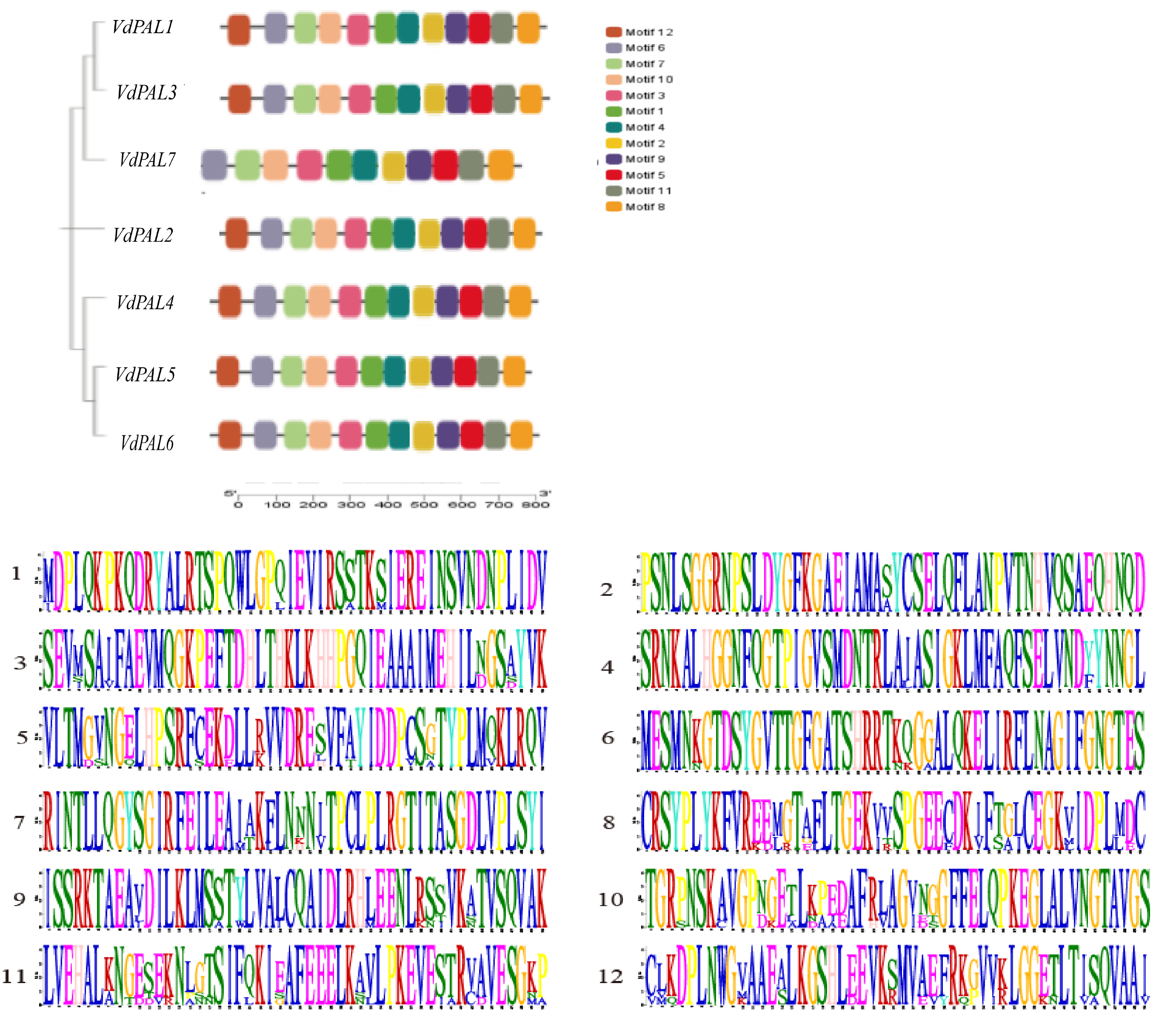

Supplement: Supplementary file 1 [file DataSheet1.zip › picture/motif.png]

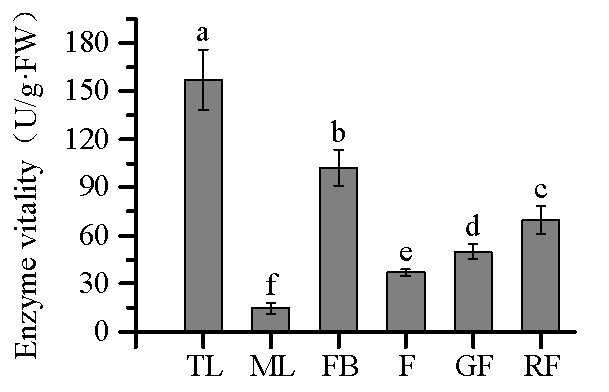

Supplement: Supplementary file 1 [file DataSheet1.zip › picture/PAL activity.png]

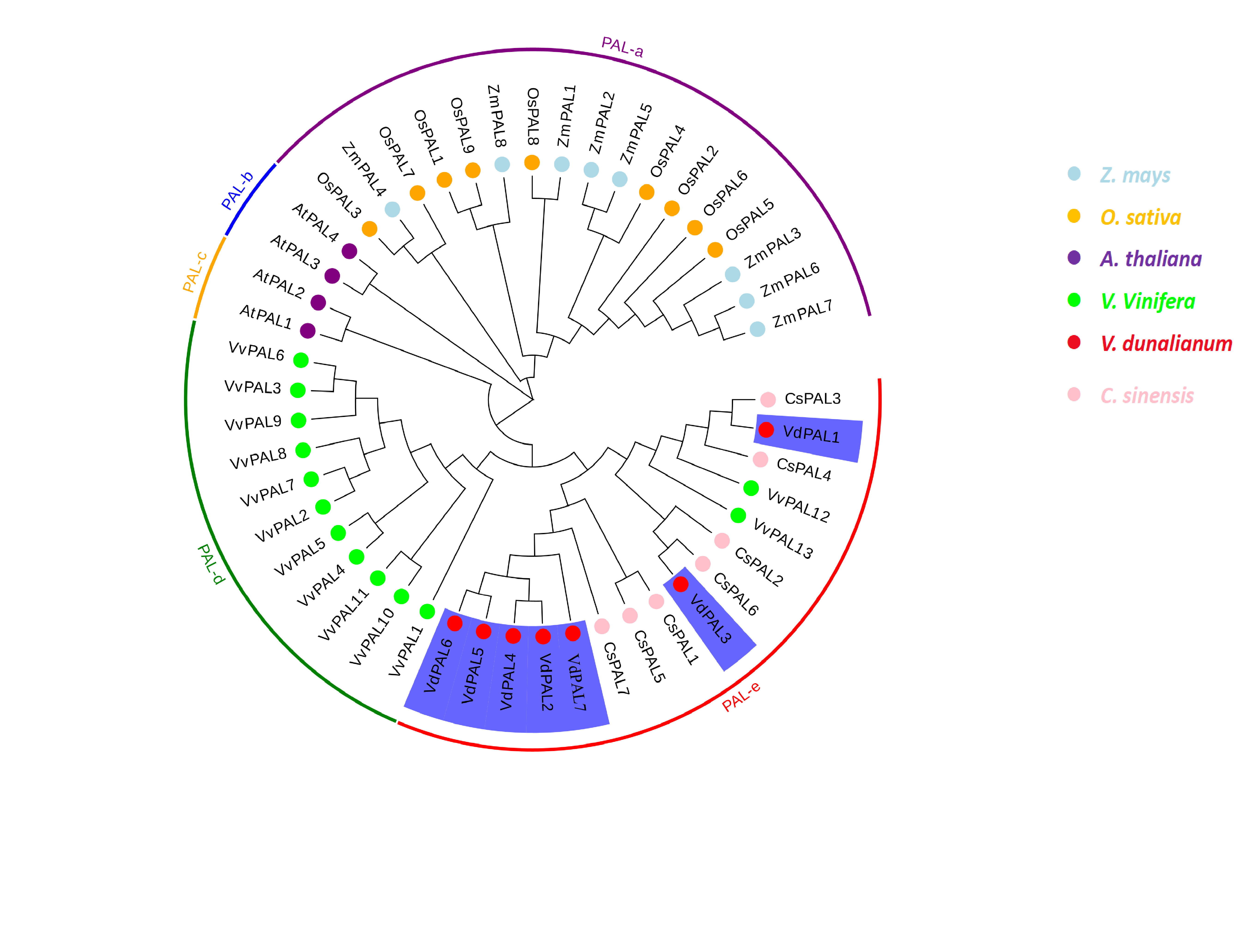

Supplement: Supplementary file 1 [file DataSheet1.zip › picture/phylogenetic tree.png]

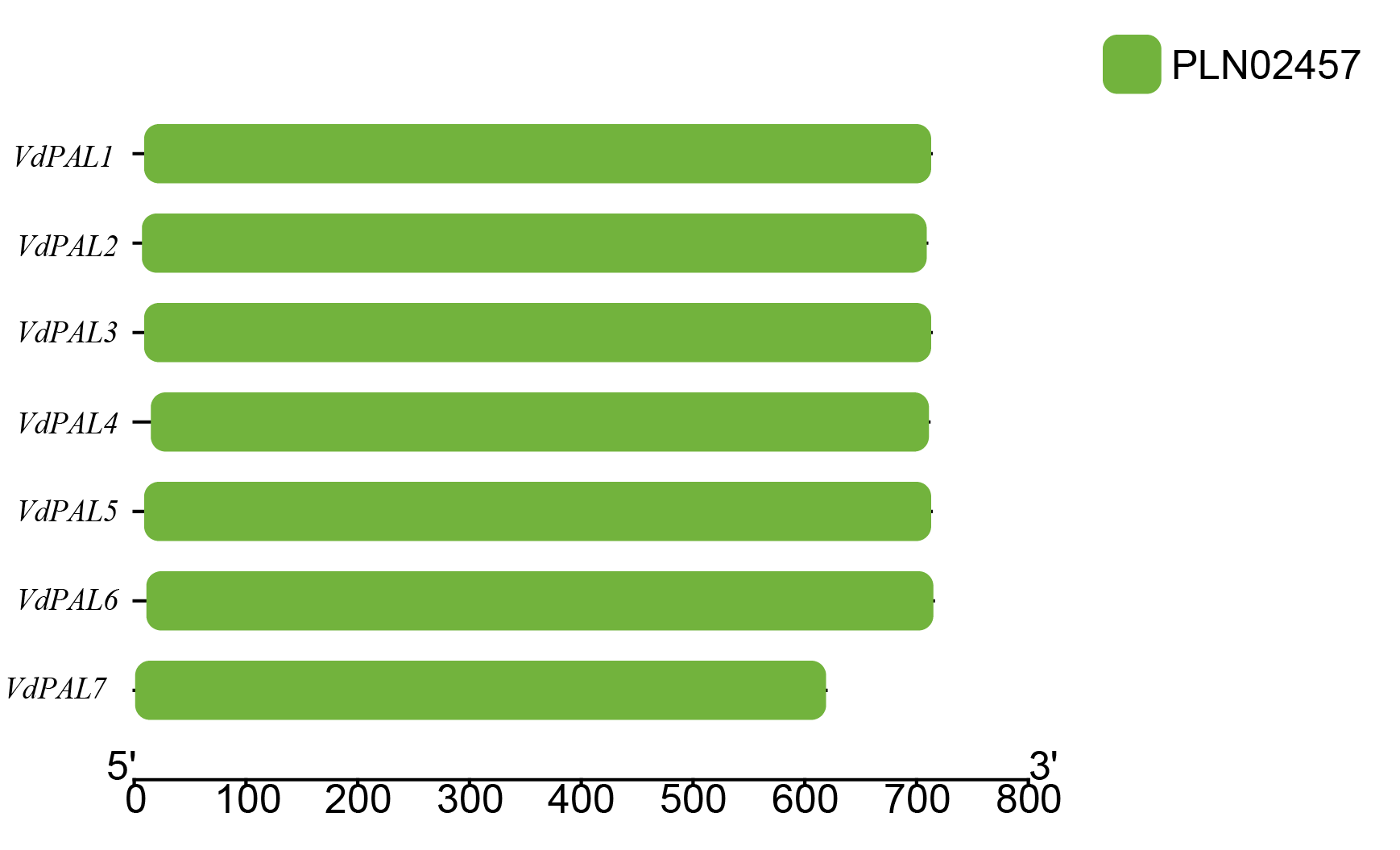

Supplement: Supplementary file 1 [file DataSheet1.zip › picture/structural domain.png]

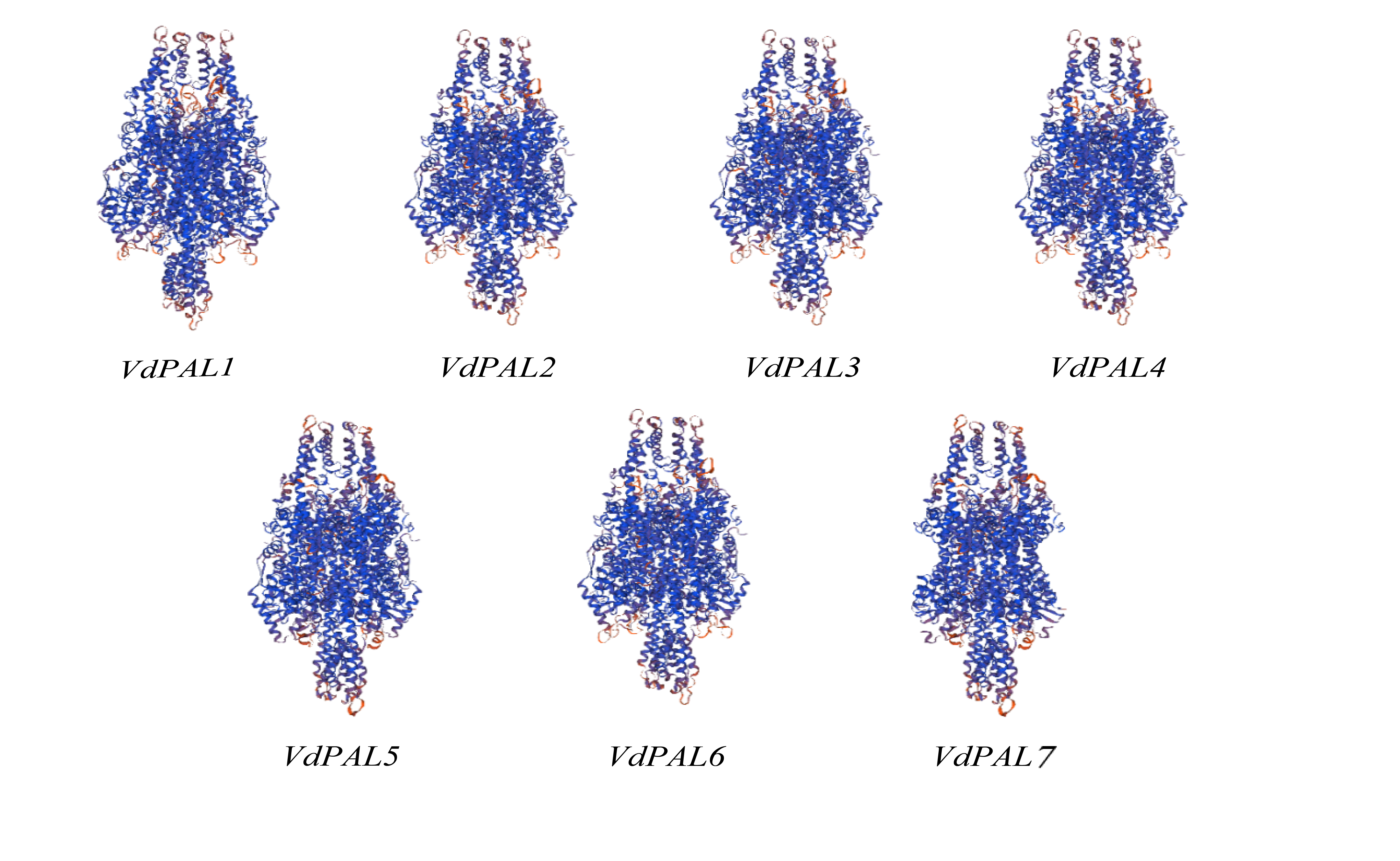

Supplement: Supplementary file 1 [file DataSheet1.zip › picture/tertiary structure of protein.png]
